# Distinctive Architecture of the Chloroplast Genome in the Chlorodendrophycean Green Algae *Scherffelia dubia* and *Tetraselmis* sp. CCMP 881

**DOI:** 10.1371/journal.pone.0148934

**Published:** 2016-02-05

**Authors:** Monique Turmel, Jean-Charles de Cambiaire, Christian Otis, Claude Lemieux

**Affiliations:** Institut de Biologie Intégrative et des Systèmes, Département de biochimie, de microbiologie et de bio-informatique, Université Laval, Québec, Québec, Canada; Austrian Federal Research Centre for Forests BFW, AUSTRIA

## Abstract

The Chlorodendrophyceae is a small class of green algae belonging to the core Chlorophyta, an assemblage that also comprises the Pedinophyceae, Trebouxiophyceae, Ulvophyceae and Chlorophyceae. Here we describe for the first time the chloroplast genomes of chlorodendrophycean algae (*Scherffelia dubia*, 137,161 bp; *Tetraselmis* sp. CCMP 881, 100,264 bp). Characterized by a very small single-copy (SSC) region devoid of any gene and an unusually large inverted repeat (IR), the quadripartite structures of the *Scherffelia* and *Tetraselmis* genomes are unique among all core chlorophytes examined thus far. The lack of genes in the SSC region is offset by the rich and atypical gene complement of the IR, which includes genes from the SSC and large single-copy regions of prasinophyte and streptophyte chloroplast genomes having retained an ancestral quadripartite structure. Remarkably, seven of the atypical IR-encoded genes have also been observed in the IRs of pedinophycean and trebouxiophycean chloroplast genomes, suggesting that they were already present in the IR of the common ancestor of all core chlorophytes. Considering that the relationships among the main lineages of the core Chlorophyta are still unresolved, we evaluated the impact of including the Chlorodendrophyceae in chloroplast phylogenomic analyses. The trees we inferred using data sets of 79 and 108 genes from 71 chlorophytes indicate that the Chlorodendrophyceae is a deep-diverging lineage of the core Chlorophyta, although the placement of this class relative to the Pedinophyceae remains ambiguous. Interestingly, some of our phylogenomic trees together with our comparative analysis of gene order data support the monophyly of the Trebouxiophyceae, thus offering further evidence that the previously observed affiliation between the Chlorellales and Pedinophyceae is the result of systematic errors in phylogenetic reconstruction.

## Introduction

The Chlorodendrophyceae is a small class of green algae belonging to the Chlorophyta that comprises marine and freshwater scaly quadriflagellates of the genera *Tetraselmis* and *Scherffelia* [1, 2]. Traditionally classified within the order Chlorodendrales of the Prasinophyceae [3, 4], this group is no longer considered to be a prasinophyte lineage, as phylogenetic analyses (based on the 18S rRNA gene and/or a few other genes) with a broad sampling of chlorophytes revealed that it is nested within a robustly supported assemblage also including the Pedinophyceae, Trebouxiophyceae, Ulvophyceae and Chlorophyceae [5–11]. But, because conflicting topologies were recovered, the branching order of the Chlorodendrophyceae and of the other classes of this large clade, called core Chlorophyta, remains uncertain. The use of a phycoplast to mediate cell division is thought to be an early innovation that took place during the evolution of the core chlorophytes: like prasinophytes, the Pedinophyceae lack a phycoplast and it is considered that the Ulvophyceae secondarily lost it [1, 8, 12]. Consistent with the phylogenetic distribution of this ultrastructural feature, phylogenetic analyses of nuclear and chloroplast rDNA operons resolved the Pedinophyceae as the earliest-diverging lineage of the core Chlorophyta, followed by the Chlorodendrophyceae, the Trebouxiophyceae and the two other classes [8].

With the goal of clarifying the relationships between the main lineages of the core Chlorophyta, we set out to sequence the chloroplast genomes of *Scherffelia dubia* and *Tetraselmis* sp. CCMP 881 and use the encoded genes to conduct phylogenomic analyses. The complete chloroplast genome sequences of about 60 chlorophytes are currently available in the reference sequence project of NCBI (as of November 2015); however, only partial genomic data (i.e. the sequences of 11 genes) have been reported for the Chlorodendrophyceae [9]. A recent phylogenomic study of 79 concatenated chloroplast genes from 61 chlorophytes representing the Pedinophyceae, Trebouxiophyceae, Ulvophyceae (Ulvales-Ulotrichales) and Chlorophyceae identified the Chlorellales (Trebouxiophyceae) + Pedinophyceae as the most basal clade of the core chlorophytes, suggesting that the Trebouxiophyceae is composed of two main clades and is thus not monophyletic [13]. An independent analysis of a 79-gene data set, in which the 44 sampled chlorophytes included representatives of an additional order of the Ulvophyceae (Bryopsidales), was in agreement with the latter observations and in addition supported the non-monophyly of the Ulvophyceae [6]. Considering that some of the deepest nodes in the trees inferred in both studies received relatively weak support and also that phylogenomic analyses are susceptible to systematic errors [14], definitive conclusions about the monophyletic status of the Trebouxiophyceae and Ulvophyceae and their relationships with the other classes of the core Chlorophyta require further analyses using expanded taxon sampling and improved models of sequence evolution.

Another important goal of the present study was to enhance our understanding of the evolutionary history of the chloroplast genome in the Chlorophyta by comparing the *Scherffelia* and *Tetraselmis* chloroplast DNAs (cpDNAs) with one another and with their chlorophyte homologs. Because the chloroplast genomes of prasinophytes belonging to the *Nephroselmis* and *Pyramimonas* genera highly resemble those of most streptophytes at the structural and gene organizational levels [15–17], it can be inferred that the common ancestor of all chlorophytes shared with streptophytes a very similar chloroplast genome architecture that is characterized by two copies of a large inverted repeat (IR) separated by small and large single-copy regions (SSC and LSC regions) that have also retained similar gene contents. But multiple losses of the IR and considerable genomic rearrangements, including frequent IR expansions/contractions and changes in the partitioning of genes between the single copy regions, took place during chlorophyte evolution, notably within the Trebouxiophyceae [15, 16, 18–24]. Consequently, on the basis of the currently available chloroplast genomes, it is difficult to infer the precise architecture of the chloroplast genome in the common ancestor of all core chlorophytes. As the Chlorodendrophyceae is likely an early-diverging lineage within the core chlorophytes [8, 11], we expected that our comparative analysis of the *Scherffelia* and *Tetraselmis* cpDNAs would provide useful information on this ancestral condition.

We report here that the quadripartite structure of the *Scherffelia* and *Tetraselmis* chloroplast genomes is unusual in displaying a SSC region that is highly reduced in size and contains no genes. The two chlorodendrophycean genomes differ by numerous rearrangements but reveal affinities with their counterparts in the Pedinophyceae and deep-diverging lineages of the Trebouxiophyceae at the levels of gene organization and gene partitioning between the IR and LSC regions. Although our phylogenomic analyses of nucleotide and amino acid data sets were plagued by conflicting topologies, they support the notion that the Chlorodendrophyceae is a deep-diverging core chlorophyte lineage and in agreement with gene order data, some of the inferred trees suggest that the Trebouxiophyceae is monophyletic.

## Materials and Methods

### Strain, Culture and DNA Extraction

*Tetraselmis* sp. CCMP 881 was obtained from the Bigelow National Center for Marine Algae and Microbiota (Maine, USA) and cultured in K medium [25], whereas *Scherffelia dubia* SAG 17.86 was obtained from the Culture Collection of Algae at the University of Goettingen and cultured in medium C [26]. Total cellular DNA was extracted as described in Turmel et al [27] and A+T-rich organellar DNA was separated from nuclear DNA by CsCl-bisbenzimide isopycnic centrifugation [15].

### Genome Sequencing, Assembly and Annotation

Sanger DNA sequencing was carried out from random clone libraries of the A+T-rich DNA fractions. Random clone libraries were prepared from 1500-2000-bp fragments derived from the A+T rich DNA fractions using the pSMART-HCKan (Lucigen Corporation, Middleton, WI) plasmid. Positive clones were selected by hybridization of each plasmid library with the original DNA used for cloning. DNA templates were amplified using the Illustra TempliPhi Amplification Kit (GE Healthcare, Baie d’Urfé, Canada) and sequenced with the PRISM BigDye terminator cycle sequencing ready reaction kit (Applied Biosystems, Foster City, CA) on Applied Biosystems model 3130XL DNA sequencers, using SR2 and SL1 primers as well as oligonucleotides complementary to internal regions of the plasmid DNA inserts (all oligonucleotide primers employed in this study are listed in S1 Table). The resulting sequences were edited and assembled using Sequencher 5.1 (Gene Codes Corporation, Ann Arbor, MI) and genomic regions not represented in the assemblies were sequenced from polymerase chain reaction (PCR)-amplified fragments using primers specific to the flanking contigs (see S1 Table for the list of oligonucleotide primers employed in this study).

Genes and open reading frames (ORFs) were identified on the final assemblies using a custom-built suite of bioinformatics tools allowing the automated execution of the following three steps: (1) ORFs were found using GETORF in EMBOSS [28], (2) their translated products were identified by BlastP [29] searches against a local database of cpDNA-encoded proteins or the nr database at the National Center for Biotechnology Information (http://www.ncbi.nlm.nih.gov/BLAST/), and (3) consecutive 100 bp segments of the genome sequence were analyzed with BlastN and BlastX [29] to identify gene sequences. Genes coding for tRNAs were independently localized using tRNAscan-SE [30]. Intron boundaries were determined by modeling intron secondary structures [31, 32] and by comparing intron-containing genes with intronless homologs. The secondary structure of the *Scherffelia* RNase P RNA was modeled according to that of the *Escherichia coli* RNA [33] and was compared to the model reported for its *Nephroselmis olivacea* homolog [34]. Circular genome maps were drawn with OGDraw [35]. To estimate the proportion of repeated sequences in the *Tetraselmis* and *Scherffelia* genomes, repeats with a minimal size of 30 bp were retrieved using REPFIND of the REPuter2.74 program [36] with the options -f -p -l -allmax and were then masked on the genome sequences using RepeatMasker (http://www.repeatmasker.org/) running under the Crossmatch search engine (http://www.phrap.org/).

### Analyses of Gene Organization

The *Tetraselmis* and *Scherffelia* chloroplast genomes were aligned using Mauve 2.3.1 [37] after removal of one copy of the IR. The number of reversals separating these genomes was estimated with GRIMM 2.01 [38]. We used a custom-built script to identify the regions that display the same gene order in the two chlorodendrophycean genomes. This Perl script employs a concatenated list of signed gene orders in the compared genomes as input file (i.e. taking into account gene polarity) and interacts with MySQL database tools (https://www.mysql.com) to perform the sorting and classification of the gene pairs. The same program was also employed to convert gene order in each of 21 selected chlorophyte cpDNAs to all possible pairs of signed genes. The presence/absence of signed gene pairs in three or more genomes were coded as binary characters using Mesquite 3.04 [39]. Losses of ancestral gene pairs were identified by tracing these characters on tree topologies with MacClade 4.08 [40] under the Dollo principle of parsimony.

### Phylogenomic Analyses

The GenBank accession numbers of the 71chloroplast genomes that were used to generate the analyzed amino acid and nucleotide data sets are given in S2 Table. The amino acid data set (PCG-AA) was assembled from the following 79 protein-coding genes: *accD*, *atpA*, *B*, *E*, *F*, *H*, *I*, *ccsA*, *cemA*, *chlB*, *I*, *L*, *N*, *clpP*, *cysA*, *T*, *ftsH*, *infA*, *minD*, *petA*, *B*, *D*, *G*, *L*, *psaA*, *B*, *C*, *I*, *J*, *M*, *psbA*, *B*, *C*, *D*, *E*, *F*, *H*, *I*, *J*, *K*, *L*, *M*, *N*, *T*, *Z*, *rbcL*, *rpl2*, *5*, *12*, *14*, *16*, *19*, *20*, *23*, *32*, *36*, *rpoA*, *B*, *C1*, *C2*, *rps2*, *3*, *4*, *7*, *8*, *9*, *11*, *12*, *14*, *18*, *19*, *tufA*, *ycf1*, *3*, *4*, *12*, *20*, *47*, *62*. It was prepared as follows: the deduced amino acid sequences from the 79 individual genes were aligned using MUSCLE 3.7 [41], the ambiguously aligned regions in each alignment were removed using TrimAl 1.3 [42] with the options block = 6, gt = 0.7, st = 0.005 and sw = 3, and the protein alignments were concatenated using Phyutility 2.2.6 [43].

Phylogenies were inferred from the PCG-AA data set using the maximum likelihood (ML) and Bayesian methods. ML analyses were carried out using RAxML 8.2.3 [44] and the GTR+Γ4 model of sequence evolution; in these analyses, the data set was partitioned by gene, with the model applied to each partition. Confidence of branch points was estimated by fast-bootstrap analysis (f = a) with 100 replicates. Bayesian analyses were performed with PhyloBayes 4.1 [45] using the site-heterogeneous CAT+Γ4 model [46]. Five independent chains were run for 10,000 cycles and consensus topologies were calculated from the saved trees using the BPCOMP program of PhyloBayes after a burn-in of 2000 cycles. Under these conditions, the largest discrepancy observed across all bipartitions in the consensus topologies (maxdiff) was 0.06, indicating that convergence between the chains was achieved. PhyloBayes analyses were also carried out using the site-heterogeneous CATGTR+Γ4 model [46] but the chains failed to converge after several weeks of computation (maxdiff = 1), indicating that at least one of the chains was stuck in a local maximum.

Four nucleotide data sets were constructed: PCG12 (first and second codon positions of the 79 protein-coding genes abovementioned), PCG12RNA (first and second codon positions of the 79 protein-coding genes plus three rRNA genes and 26 tRNA genes), PCG123degen (all degenerated codon positions of the 79 protein-coding genes), and PCG123degenRNA (all degenerated codon positions of the 79 protein-coding genes plus three rRNA genes and 26 tRNA genes). The PCG12 and PCG123degen data sets were prepared as follows. The multiple sequence alignment of each protein was converted into a codon alignment, the poorly aligned and divergent regions in each codon alignment were excluded using Gblocks 0.91b [47] with the -t = c, -b3 = 5, -b4 = 5 and -b5 = half options, and the individual gene alignments were concatenated using Phyutility 2.2.6 [43]. The third codon positions of the resulting PCG123 alignment were excluded using Mesquite 3.04 [39] to produce the PCG12 data set, and the Degen1.pl 1.2 script of Regier et al. [48] was applied to the same concatenated alignment to generate the PCG123degen data set.

To obtain the PCG12RNA and PCG123degenRNA data sets, the PCG12 and PCG123degen matrices were each merged with the concatenated alignment of the following RNA genes: *rrf*, *rrl*, *rrs*, *trnA*(ugc), *C*(gca), *D*(guc), *E*(uuc), *F*(gaa), *G*(gcc), *G*(ucc), *H*(gug), *I*(cau), *I*(gau), *K*(uuu), *L*(uaa), *L*(uag), *Me*(cau), *Mf*(cau), *N*(guu), *P*(ugg), *Q*(uug), *R*(acg), *R*(ucu), *S*(gcu), *S*(uga), *T*(ugu), *V*(uac), *W*(cca), *Y*(gua). The latter genes were aligned using MUSCLE 3.7 [41], the ambiguously aligned regions in each alignment were removed using TrimAl 1.3 [42] with the options block = 6, gt = 0.9, st = 0.4 and sw = 3, and the individual alignments were concatenated using Phyutility 2.2.6 [43].

ML analyses of the nucleotide data sets were carried out using RAxML 8.2.3 [44] and the GTR+Γ4 model of sequence evolution. Each data set was partitioned into gene groups, with the model applied to each partition. The partitions used for the PCG12 and PCG123degen data sets included the 79 individual protein-coding genes, while those used for the PCG12RNA and PCG123degenRNA data sets included two RNA gene groups (the concatenated rRNA genes and the concatenated tRNA genes) in addition to the latter protein-coding gene partitions. Confidence of branch points was estimated by fast-bootstrap analysis (f = a) with 100 replicates.

## Results and Discussion

### The *Scherffelia* and *Tetraselmis* Chloroplast Genomes Resemble Their Core Chlorophyte Counterparts at Several Levels

**Fig 1:**
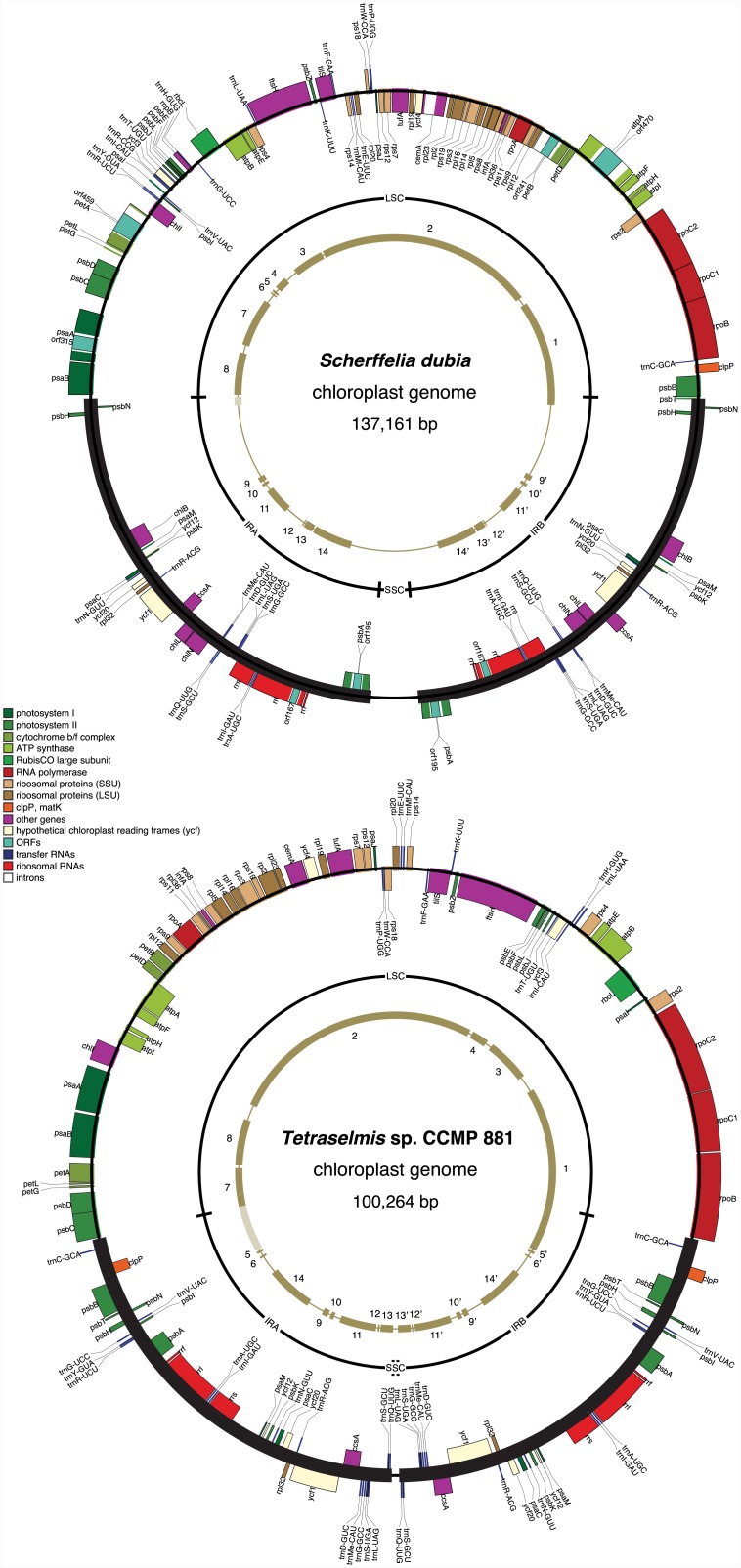
Gene maps of the *Scherffelia* and *Tetraselmis* chloroplast genomes. Filled boxes represent genes, with colors denoting gene categories as indicated in the legend. Genes on the outside of each map are transcribed counterclockwise; those on the inside are transcribed clockwise. The second outermost middle ring indicates the positions of the IR, LSC and SSC regions. Thick lines in the innermost ring represent the gene clusters conserved between the two chlorodendrophycean cpDNAs.

The *Scherffelia* and *Tetraselmis* chloroplast genomes were assembled as circular-mapping and IR-containing molecules of 137,161 bp [GenBank:KU167098] and 100,264 bp [GenBank:KU167097], respectively (Fig 1). The assembly of the *Scherffelia* genome includes a total of 585 reads (from 330 individual clones and 17 PCR fragments) with an average length of 798 bp and that of the *Tetraselmis* genome a total of 651 reads (from 564 individual clones and three PCR fragments) with an average length of 855 bp. The general features of both chlorodendrophycean genomes are compared with those previously reported for selected core chlorophytes in Table 1. Their sizes are within the lower range found for their counterparts—genome size of core chlorophytes varies from 94,206 bp in the core trebouxiophycean *Choricystis minor* [18] to 521,168 bp in the chlorophycean *Floydiella terrestris* [19]–and their AT contents also fall within the reported limits, from 42.3% in the core trebouxiophycean Trebouxiophyceae sp. MX-AZ01 [49] to 72.8% in the chlorophycean *Schizomeris leibleinii* [50]. About 60% of the 37-kb increased size of the *Scherffelia* cpDNA relative to its *Tetraselmis* homolog is attributable to an enlarged IR; the remaining fraction is accounted for by longer intergenic regions (i.e. a lower gene density), the presence of five extra genes, and the occurrence of seven introns (Table 1 and Fig 1). Variations in IR size, gene density, and number of introns are common within the major groups of core chlorophytes [6, 15, 16, 18–20, 23, 24].

**Table 1:** General features of *Scherffelia*, *Tetraselmis* and other core chlorophyte chloroplast genomes.

Taxon	A+T	Size (bp)			Genes ^a^		Introns ^b^			Repeats ^c^
	(%)	Genome	IR	SSC	No.	%	GI	GII	%	(%)
**Chlorodendrophyceae**										
*Scherffelia dubia*	67.4	137,161	32,310	3,385	104	58.5	3	4	8.4	0.3
*Tetraselmis* sp. CCMP 881	66.0	100,264	21,342	392	99	76.5				0
**Pedinophyceae**										
*Marsupiomonas* sp. NIES 1824	59.7	94,262	9,926	6,225	105	75.3				0.3
*Pedinomonas tuberculata*	66.6	126,694	16,074	7,927	106	55.8	5	5	9.9	1.9
**Chlorellales**										
*Parachlorella kessleri*	70.0	123,994	10,913	13,871	112	63.3	1		0.2	4.0
*Pseudochloris wilhelmii*	63.3	109,775	12,798	17,968	113	74.1	1		0.2	4.2
**Core Trebouxiophyceae**										
*Geminella terricola*	67.3	187,843	18,786	10,954	109	42.5	1	1	1.0	22.7
*"Koliella" corcontica*	72.0	117,543	15,891	8,415	105	61.8	8		12.3	11.6
*Planctonema lauterbornii*	66.8	114,128	10,577	11,068	111	67.1	1		0.2	7.3
*"Chlorella" mirabilis*	68.5	167,972	6,835	33,215	110	47.6				5.5
*Parietochloris pseudoalveolaris*	68.4	145,947	6,786	16,399	109	52.5				10.2
**Ulvophyceae**										
*Oltmannsiellopsis viridis*	59.5	151,933	18,510	33,610	104	53.5	5		6.8	11.1
*Pseudendoclonium akinetum*	68.5	195,867	6,039	42,875	105	43.2	27		15.3	5.3
*Bryopsis plumosa*	69.2	106,859			108	61.9	7	6	8.3	2.4
**Chlorophyceae**										
*Oedogonium cardiacum*	70.5	196,547	35,492	45,200	99	52.6	17	4	17.9	1.3
*Acutodesmus obliquus*	73.1	161,452	12,023	64,967	97	56.1	7	2	7.9	2.6
*Chlamydomonas reinhardtii*	65.5	203,826	22,211	78,099	94	44.1	5	2	6.8	16.5

^a^ Intronic genes and freestanding ORFs not usually found in green plant chloroplast genomes are not included in these values. Duplicated genes were counted only once. The proportion of coding sequences in the genome is also provided.

^b^ Number of group I (GI) and group II (GII) introns is given. The proportion of intron sequences in the genome is also provided.

^c^ Nonoverlapping repeat elements were mapped on each genome with RepeatMasker using as input sequences the repeats of at least 30 bp identified with REPuter. The proportion of the estimated repeat sequences in the genome is given.

**Fig 2:**
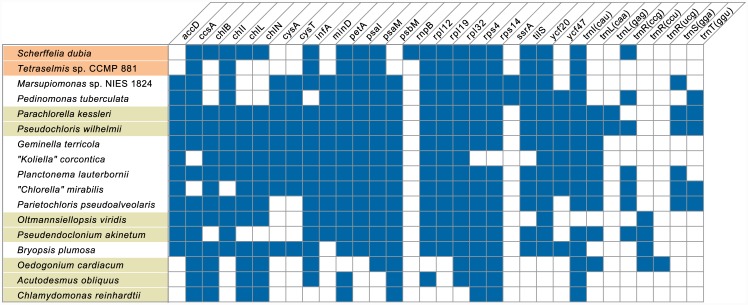
Gene repertoires of the chloroplast genomes compared in this study. Only the conserved genes that are missing in one or more genomes are indicated. The presence of a gene is denoted by a blue box. A total of 85 genes are shared by all compared genomes: *atpA*, *B*, *E*, *F*, *H*, *I*, *cemA*, *clpP*, *ftsH*, *petB*, *D*, *G*, *L*, *psaA*, *B*, *C*, *J*, *psbA*, *B*, *C*, *D*, *E*, *F*, *H*, *I*, *J*, *K*, *L*, *N*, *T*, *Z*, *rbcL*, *rpl2*, *5*, *14*, *16*, *20*, *23*, *36*, *rpoA*, *B*, *C1*, *C2*, *rps2*, *3*, *7*, *8*, *9*, *11*, *12*, *18*, *19*, *rrf*, *rrl*, *rrs*, *tufA*, *ycf1*, *3*, *4*, *12*, *trnA*(ugc), *C*(gca), *D*(guc), *E*(uuc), *F*(gaa), *G*(gcc), *G*(ucc), *H*(gug), *I*(gau), *K*(uuu), *L*(uaa), *L*(uag), *Me*(cau), *Mf*(cau), *N*(guu), *P*(ugg), *Q*(uug), *R*(acg), *R*(ucu), *S*(gcu), *S*(uga), *T*(ugu), *V*(uac), *W*(cca), *Y*(gua).

Similarities of the *Scherffelia* and *Tetraselmis* chloroplast genomes to other core chlorophyte cpDNAs extend to the complement of conserved genes (Fig 2), which varies in number from 94 in the chlorophyceans *Chlamydomonas reinhardtii* and *Volvox carteri* to 114 in the closely related core trebouxiophyceans *Coccomyxa subellipsoidea*, *Paradoxia multiseta* and Trebouxiophyceae sp. MX-AZ01. The 104 conserved genes in the *Scherffelia* cpDNA code for 73 proteins and 31 RNA species, i.e. three rRNAs (*rrs*, *rrl* and *rrf*), 27 tRNAs (*trn* genes) that can read all codons present in the genome, and the RNA subunit of RNase P (*rnpB*). The latter RNA species shares 36.6% sequence identity with its homolog in the prasinophyte *Nephroselmis olivacea* and displays the typical secondary structural elements reported for RNase P RNA subunits (S1 Fig). Relative to the *Scherffelia* cpDNA, the *Tetraselmis* genome is lacking three genes encoding proteins essential for chlorophyll synthesis in the dark (*chlB*, *chlL* and *chlN*) as well as *trnR*(ccg) and *rnpB*. These five genes are absent from the chloroplast genomes of other core chlorophytes and a number of prasinophytes [15, 18]. The *chl* genes most probably completely vanished from *Tetraselmis*, because Blastp searches of the transcriptome shotgun assembly protein database of NCBI (tsa_nr) using the *Scherffelia chlB*, *chlL* and *chlN* sequences as queries revealed no significant similarity with the transcriptome of the halophilic microalga *Tetraselmis* sp. GSL018 which is included in this database. Both *Scherffelia* and *Tetraselmis* are missing six protein-coding genes that are present in other core chlorophytes (Fig 2), suggesting that losses of these genes occurred before the emergence of the Chlorodendrophyceae. BlastP searches of the tsa_nr database of NCBI using as queries the proteins encoded by the corresponding *Pedinomonas minor* genes identified three sequences in the *Tetraselmis* sp. GSL018 transcriptome: JAC75372 (AccD query, *E* = 4e-11), JAC66565 (CysA query, *E* = 1e-28) and JAC64732 (PsbM query, *E* = 9e-08). JAC64732 was confirmed to be the genuine PsbM (an essential component of the photosystem II) in BlastP searches of the nr database and consistent with this result, a subcellular localization analysis using TargetP [51] strongly predicted (score of 0.942) that it contains a chloroplast transit peptide with a presequence length of 52 residues. In contrast, the JAC75372 and JAC66565 sequences showed no clear similarity to the chloroplast-encoded *accD* and *cysA* gene products and TargetP predicted the presence of an N-terminal mitochondria-targeting signal in each protein. Hence, although it remains to be confirmed that *psbM* is lacking in the chloroplast genome of *Tetraselmis* sp. GSL018, our results support the notion that this gene migrated to the nucleus before the emergence of the Chlorodendrophyceae. In prasinophytes, *psbM* disappeared from the chloroplast on three independent occasions [15] and was also shown to be nuclear-encoded in the Mamiellophyceae [52].

While the *Tetraselmis* chloroplast genome is lacking introns, seven are found in the *Scherffelia* genome (Fig 1 and Table 2). Three group I introns with internal ORFs coding for putative homing endonucleases are inserted within *psaA*, *psbA* and *rrl* at positions that have been previously reported for other core chlorophytes [18, 19, 23, 24] and for the prasinophyte *Monomastix* [16]. Four group II introns, three of which encode putative proteins with reverse-transcriptase and intron maturase activities in their domain IV, interrupt *atpA*, *cemA*, *petA* and *petB*; only the insertion site of the *petB* intron has been previously identified in a green alga, i.e. the core trebouxiophycean *Watanabea reniformis* [18]. Sequence alignments and structural comparisons of these introns revealed strong similarities between the *atpA* and *cemA* introns and between the *petA* and *petB* introns (S2 Fig). The latter introns are also similar to the group II intron found in the *psbA* gene of *Euglena myxocylindracea* [53].

**Table 2:** Introns in the *Scherffelia* chloroplast genome.

		Intron ORF		
Intron designation ^a^	Subgroup ^b^	Location ^c^	Type ^d^	Size (codons)
**Group I introns**				
*psaA* 1601	IB4	L8	LAGLIDADG (2)	315
*psbA* 525	IA2	L6	GIY-YIG	195
*rrl* 2593	IA3	L6	LAGLIDADG (1)	167
**Group II introns**				
*atpA* 441	IIB	Domain IV	RT-X	470
*cemA* 17	IIB	–	–	–
*petA* 116	IIB	Domain IV	RT-X	459
*petB* 24	IIB	Domain IV	RT-X	241

^a^ The insertion sites of the introns in protein-coding genes are given relative to the corresponding genes in *Mesostigma* cpDNA whereas the insertion site of the *rrl* intron is given relative to the *E*. *coli* 23S rRNA. For each insertion site, the position corresponds to the nucleotide immediately preceding the intron.

^b^ Group I introns were classified according to Michel and Westhof [31], whereas classification of group II introns was according to Michel et al. [32].

^c^ L followed by a number refers to the loop extending the base-paired region identified by the number; Domain refers to a domain of the group II intron secondary structure.

^d^ For the group I intron ORFs, the conserved motif in the predicted homing endonuclease is given, with the number of copies of the LAGLIDADG motif indicated in parentheses. For the group II intron ORFs, RT and X refer to the reverse transcriptase and maturase domains, respectively.

### Both Chlorodendrophycean Chloroplast Genomes Feature an Unusual Quadripartite Structure

Unlike all IR-containing chlorophyte genomes that have been examined so far, the *Scherffelia* and *Tetraselmis* cpDNAs exhibit no genes in their SSC region (Fig 1). At 3,385 bp and 392 bp, respectively, the *Scherffelia* and *Tetraselmis* SSC regions are the shortest among all completely sequenced IR-containing chlorophyte cpDNAs (Table 1). Prior to our study, the SSC regions of the pedinophyceans *Pedinomonas minor*, *Pedinomonas tubercula* and *Marsupiomonas* sp., which range from 6,225 to 7,927 bp and encode eight or nine conserved genes, were known to have the smallest sizes [18, 20]. To our knowledge, no chloroplast genome has previously been reported to harbor a SSC region devoid of any gene. Although the genome of the streptophyte green alga *Klebsormidium flaccidum* shares a greatly reduced SSC (1,817 bp) with its chlorodendrophycean homologs, it has retained the *ccsA* gene [54]. Conceptually, the chloroplast genome of the alveolate *Chromera velia*, which adopts a linear conformation with terminal inverted repeats [55], could be viewed as an extreme case of IR expansion toward the SSC region and according to this hypothesis, complete loss of the *Chromera* SSC region would have occurred concomitantly with the linearization of the genome. However, the situation differs in the Chlorodendrophyceae, as both the *Tetraselmis* and *Scherffelia* genomes adopt a circular conformation. There is no doubt that these two green algal genomes are circular-mapping molecules considering that we obtained several plasmid clones and individual sequence reads extending over both IR/SSC junctions of *Tetraselmis* and that we recovered independent PCR fragments and several sequence reads spanning both IR/SSC junctions of *Scherffelia*.

**Fig 3:**
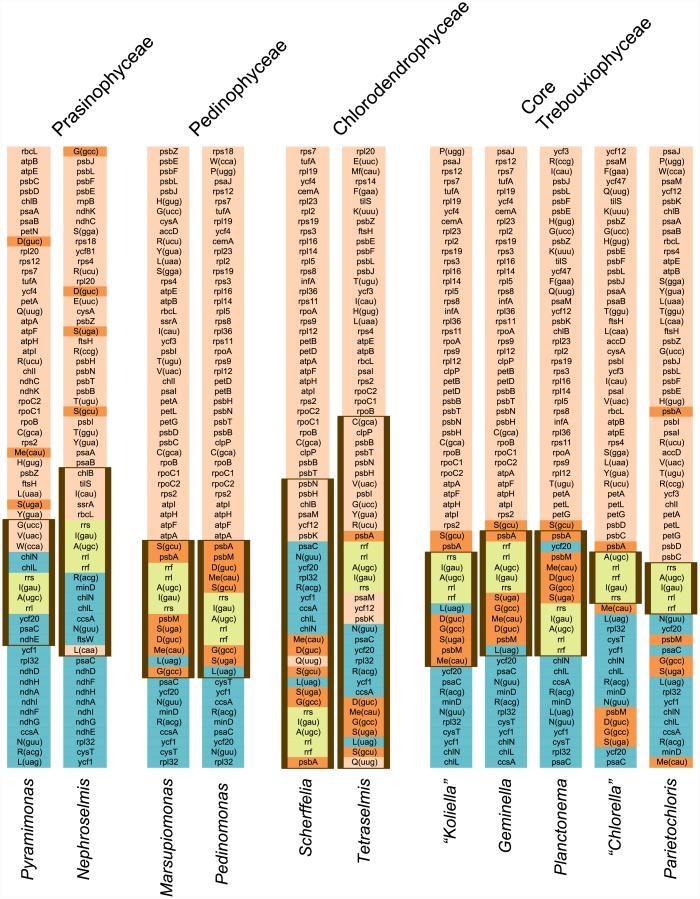
Gene partitioning patterns of the *Scherffelia*, *Tetraselmis* and other chlorophyte chloroplast genomes. For each genome, one copy of the IR (thick vertical lines) and the entire SSC region are represented, but only the portion of the LSC region in the vicinity of the IR is displayed. The five genes composing the rDNA operon are highlighted in light green. The color assigned to each of the remaining genes is dependent upon the position of the corresponding gene relative to the rDNA operon in the cpDNA of the streptophyte alga *Mesostigma viride*, a genome displaying an ancestral gene partitioning pattern [56]. The genes highlighted in blue are found within or near the SSC region in this streptophyte genome (downstream of the rDNA operon), whereas those highlighted in light orange are found within or near the LSC region (upstream of the rDNA operon). The dark orange boxes denote the genes of LSC origin that have been acquired by the IRs of core chlorophytes (pedinophyceans, chlorodendrophyceans and core trebouxiophyceans). Note that, to simplify the comparison of gene order, some genomes are represented in their alternative isomeric form as compared to that used for the genome sequence deposited in GenBank.

The lack of genes in the SSC regions of the *Scherffelia* and *Tetraselmis* cpDNAs is compensated by the rich gene complement of their IRs. Among all completely sequenced IR-containing green algal cpDNAs, the *Scherffelia* and *Tetraselmis* IRs are the most rich in conserved genes and as will be discussed below, this situation is partly due to the acquisition, through multiple IR expansions, of genes typically found in the LSC and SSC regions. In addition to the rRNA operon, the 32,310-bp IR of *Scherffelia* contains 14 protein-coding genes and nine tRNA genes, whereas the 21,342-bp pair IR of *Tetraselmis* contains 15 protein-coding genes and 14 tRNA genes (Fig 1). The six-gene difference between these IRs reflects the presence of nine genes unique to the *Tetraselmis* IR and the absence of three genes in the *Tetraselmis* IR that are found in its *Scherffelia* homolog. Four of the unique genes in the *Tetraselmis* IR are easily explained by a relatively recent IR expansion/contraction event (Fig 3) that either incorporated neighboring genes present in the *Tetraselmis* LSC or excluded the corresponding genes from the *Scherffelia* IR. From the available data, however, it is difficult to infer the events accounting for the remaining extra genes in the *Tetraselmis* IR, whose orthologs in the *Scherffelia* genome reside at two separate locations in the LSC.

The *Scherffelia* IR displays, near one of the IR/LSC boundaries, a sequence of 8,819 bp that contains no conserved genes and is missing in *Tetraselmis* (Fig 1). Its nucleotide composition is similar to that of the entire genome (66% versus 67.4% A+T). Several ORFs of more than 75 bp were found in this sequence (see [GenBank:KU167098]) but none of them disclosed significant homology to any known proteins. Long IR segments lacking conserved genes have also been observed in a number of chlorophyte chloroplast genomes [16–18, 21, 57]. In the cases of the *Oedogonium cardiacum* [21], *Pyramimonas parkeae* [16] and *Nephroselmis olivacea* [17] genomes, these segments contain ORFs that were probably acquired through horizontal gene transfers.

### Despite Their High Level of Synteny, the *Scherffelia* and *Tetraselmis* Chloroplast Genomes Display Important Rearrangements

**Fig 4:**
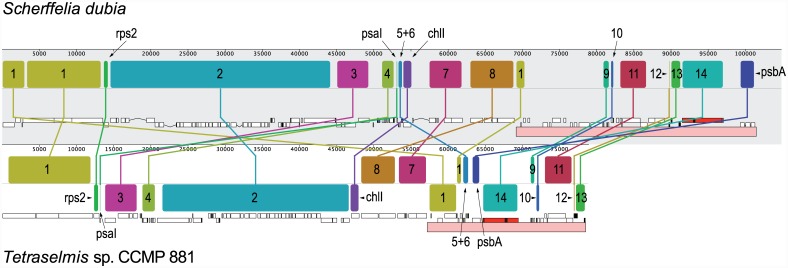
Extent of rearrangements between the *Scherffelia* and *Tetraselmis* chloroplast genomes. These genomes were aligned using Mauve 2.3.1. Only one copy of the IR (pink boxes) is shown for each genome. The blocks of colinear sequences containing two or more genes are numbered as in Fig 1. Gene clusters 5 and 6 were retrieved as a single locally colinear block because their very small sizes did not allow them to be resolved in Mauve. Conversely, the gene cluster spanning the LSC/IR junction (cluster 1) was fragmented into three colinear blocks in Mauve because only one copy of the IR was included in this analysis and also because the two genomes were treated as linear instead of circular molecules (the genomes were linearized at the LSC/IR junction).

Gene order is relatively well conserved between the *Scherffelia* and *Tetraselmis* cpDNAs, as 91 of the 99 genes they share form 14 syntenic blocks (Fig 1). Eight syntenic blocks are found in the IR alone. All blocks contain fewer than ten genes except block 2, which encodes 39 genes and is entirely comprised within the LSC. With nine genes, block 1 ranks second in term of gene number and encompasses both the LSC and IR. The extent of gene rearrangements between the two chlorodendrophycean genomes can be visualized in the Mauve genome alignment shown in Fig 4. Using GRIMM, it was estimated that a minimum of 21 reversals are required to convert the chloroplast gene order of *Scherffelia* into that of *Tetraselmis*. These results indicate that important rearrangements have occurred in both the IR and LSC regions during the evolution of the Chlorodendrophyceae.

Small repeats have been associated with cpDNA rearrangements in some land plant lineages [58, 59]. However, there is no evidence that repeated sequences account for the gene rearrangements observed in *Scherffelia* and *Tetraselmis*. Like other chlorophyte genomes with a low proportion of non-coding sequences, notably their prasinophycean and pedinophycean homologs [15, 18, 20], both chlorodendrophycean cpDNAs are very poor in small repeats (Table 1).

### Chloroplast Phylogenomic Analyses Identify the Chlorodendrophyceae as an Early Lineage of the Core Chlorophyta

**Fig 5:**
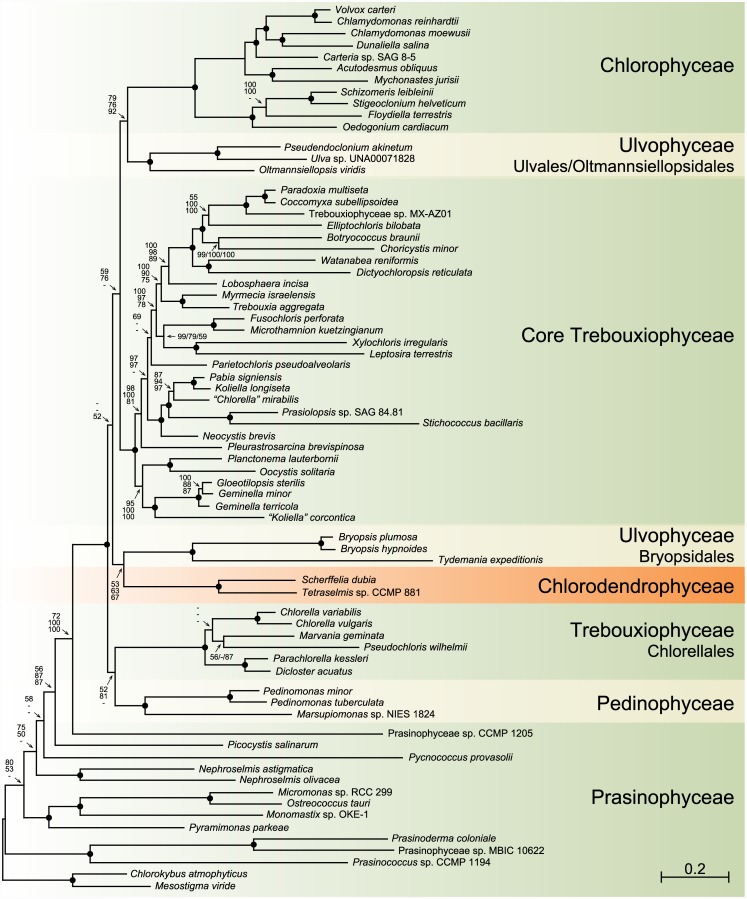
ML phylogeny of chlorophytes inferred using the amino acid and nucleotide data sets assembled from 79 protein-coding genes. The best-scoring RAxML tree inferred from the amino acid (PCG-AA) data set under the GTR+Γ4 model is presented. Bootstrap support (BS) values are reported on the nodes: from top to bottom or left to right, are shown the values for the analyses of the PCG-AA and the nucleotide PCG123degen and PCG12 data sets. A black dot indicates that the corresponding branch received a BS value of 100% in all three analyses; a dash represents a BS value < 50%. The scale bar denotes the estimated number of amino acid substitutions per site.

**Fig 6:**
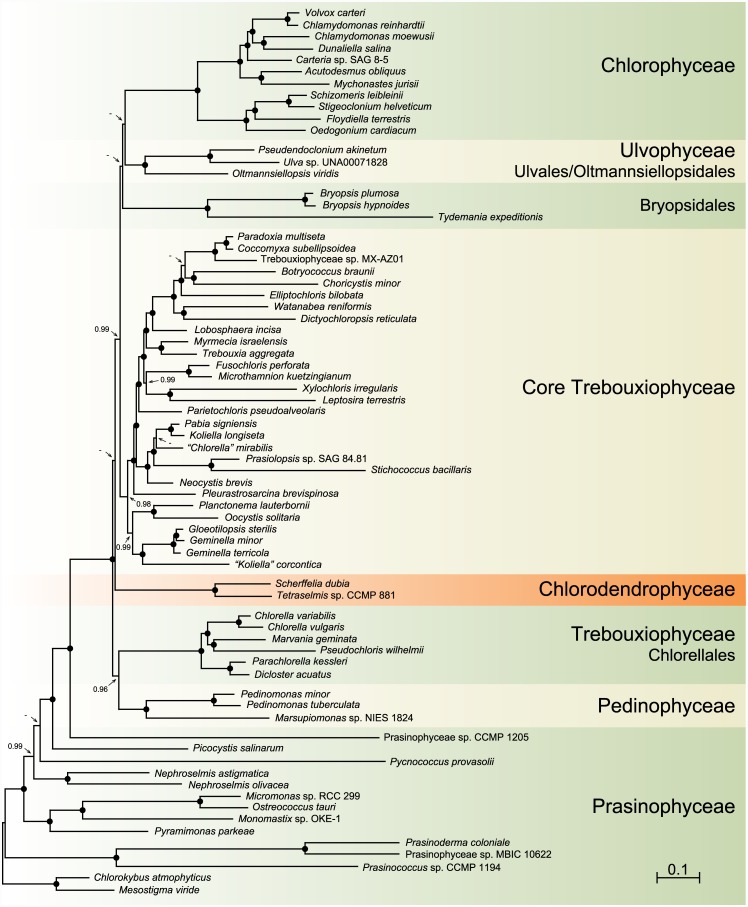
Bayesian phylogeny of chlorophytes inferred using the PCG-AA data set assembled from 79 cpDNA-encoded proteins. The majority-rule posterior consensus tree inferred with Phylobayes under the CAT+Γ4 model is presented. Posterior probability values are reported on the nodes: a black dot indicates that the corresponding branch received a value of 1.00 whereas a dash indicates a value < 0.95. The scale bar denotes the estimated number of amino acid substitutions per site.

**Fig 7:**
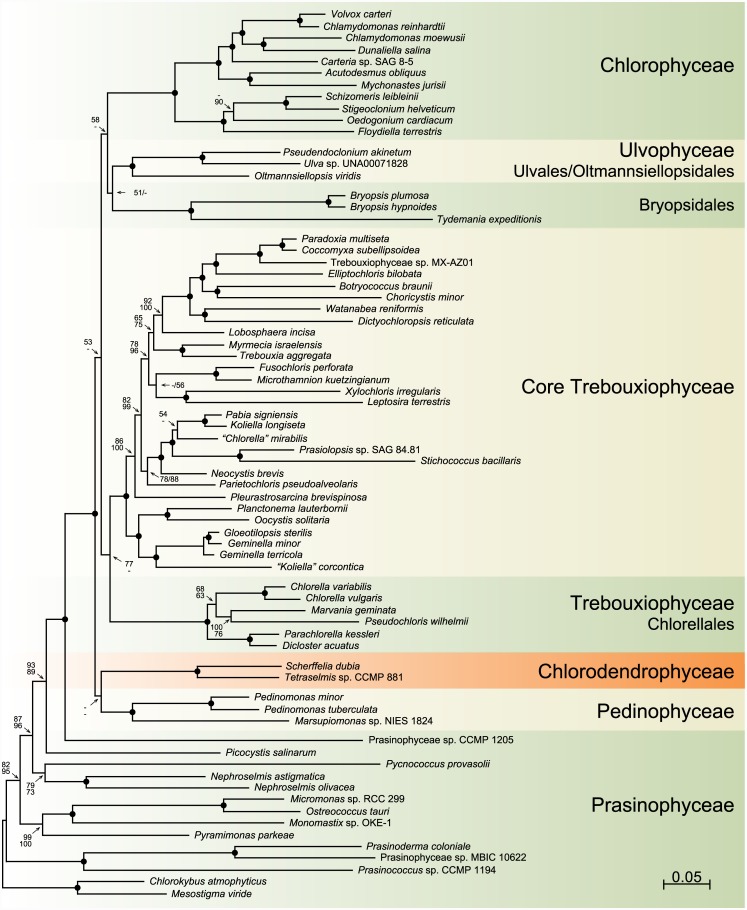
ML phylogeny of chlorophytes inferred using the nucleotide PCG12RNA and PCG123degenRNA data sets assembled from 79 protein-coding and 29 RNA-coding genes. The best-scoring RAxML tree inferred from the PCG12RNA data set under the GTR+Γ4 model is presented. BS values are reported on the nodes: from top to bottom or left to right, are shown the values for the analyses of the PCG12RNA and PCG123degenRNA data sets. A black dot indicates that the corresponding branch received a BS value of 100% in both analyses; a dash represents a BS value < 50%. The scale bar denotes the estimated number of nucleotide substitutions per site.

Before comparing the gene orders and quadripartite structures of the *Scherffelia* and *Tetraselmis* genomes with their chlorophyte counterparts, we wish to present the analyses that provide the phylogenetic context to discuss these results. Our chloroplast phylogenomic analyses were carried out using one amino acid and four nucleotide data sets, all including 71 taxa (Figs 5–7). The amino acid data set (PCG-AA, 15,350 sites) and two of the nucleotide data sets were assembled from 79 protein-coding genes; the PCG12 nucleotide data set (30,684 sites) included only the first two codon positions, whereas the PCG123degen nucleotide data set (40,026 sites) comprised all three codon positions but these were fully degenerated using degen1 [48] to reduce compositional heterogeneity while leaving the inference of nonsynonymous changes largely intact. The two remaining nucleotide data sets (PCG12RNA, 36,658 sites and PCG123degenRNA, 52,000 sites) were assembled from the 79-protein coding genes and 29 RNA-coding genes (three rRNA genes and 26 tRNA genes) using again either the first two codon positions or the degen1-degenerated nucleotides at all three codon positions. Missing data account for less than 6.1% of each data set.

The topologies we recovered are dependent upon the nature of the data set and the method of analysis employed, and they differ mainly with respect to the positioning of the major lineages of the core Chlorophyta (Figs 5–7). Analyses of the PCG-AA and nucleotide data sets derived from the 79 protein-coding genes using RAxML and the site-homogeneous GTR+ Γ4 model of sequence evolution (Fig 5) reveal identical relationships for the major lineages of core chlorophytes, with the Chlorodendrophyceae being sister to the Bryopsidales, and the Chlorodendrophyceae + Bryopsidales being sister to the core Trebouxiophyceae + Ulvales/Oltmannsiellopsidales + Chlorophyceae; however, these relationships received weak support. In the analysis of the PCG-AA data set using Phylobayes and the site-heterogeneous CAT+ Γ4 model (Fig 6), the Chlorodendrophyceae occupy the same position but the Bryopsidales diverge at the base of the Ulvales/Oltmannsiellopsidales + Chlorophyceae, the latter position being supported by low posterior probability values. In the RAxML trees inferred using the 108-gene data sets (Fig 7), the Ulvophyceae and Trebouxiophyceae each form a weakly supported monophyletic assemblage and the Chlorodendrophyceae are weakly affiliated with the Pedinophyceae, with the latter clade occupying the most basal position of the core chlorophytes.

In contrast to recent phylogenetic studies based on concatenated chloroplast protein-coding genes in which only 11 genes of *Tetraselmis* were sampled [5–7, 9], our phylogenomic analyses are congruent in supporting a basal placement of the Chlorodendrophyceae within the core Chlorophyta. *Tetraselmis* affiliated with *Oltmannsiellopsis* in two of these studies, forming either a late-diverging clade sister to the Ulvales-Ulotrichales [9] or a clade representing an early branch [7]. In the nucleotide-based trees inferred by Melton et al. [5] and by Leliaert and Lopez-Bautista [6], *Tetraselmis* was resolved as a late divergence, being positioned at the base of an ulvophycean assemblage formed by representatives of the Oltmansiellopsidales, Ulvales-Ulotrichales, Dasycladales and Trentepohliales; however, it was recovered as the earliest-diverging lineage of the core Chlorophyta in the amino-acid based trees inferred by Leliaert and Lopez-Bautista [6].

A basal placement of the Chlorodendrophyceae was also observed in the phylogeny inferred by Marin et al. [8] from complete nuclear- and chloroplast-encoded rDNA operons. Consistent with an early origin of the phycoplast, the clade formed by three *Tetraselmis* species and *Scherffelia dubia* diverged just after the Pedinophyceae and displayed a sister-relationship with respect to the Trebouxiophyceae + Ulvophyceae + Chlorophyceae. Interestingly, this relatively robust topology in which the Trebouxiophyceae and Ulvophyceae appear to be monophyletic is entirely congruent with the trees inferred here from the 108-gene data sets including 29 RNA-coding genes even though the precise positions of the Pedinophyceae and Chlorodendrophyceae in the latter trees are ambiguous (Fig 7).

### The Chloroplast Genomes of Chlorodendrophyceans and Core Chlorophytes Display Notable Similarities in Gene Organization

Despite their differences in gene content and gene organization, the *Scherffelia* and *Tetraselmis* IRs share a number of derived features with their pedinophycean and trebouxiophycean homologs, notably the presence of several genes that are encoded by the LSC region in prasinophyte genomes that have retained an ancestral quadripartite structure (Fig 3). All seven pedinophycean genes falling in this category, except *psbM* (a nuclear-encoded gene in the Chlorodendrophyceae), are found within the IRs of *Scherffelia* and *Tetraselmis*. Besides supporting the affinities of the Chlorodendrophyceae with the Pedinophyceae and Trebouxiophyceae, these observations indicate that the IR of the common ancestor of the core chlorophytes had already expanded by acquiring a set of seven genes from the LSC region. However, the exact gene organization of this ancestral IR cannot be inferred on the basis of the available data because of the great variability of this cpDNA region in the Pedinophyceae, Chlorodendrophyceae and Trebouxiophyceae.

**Fig 8:**
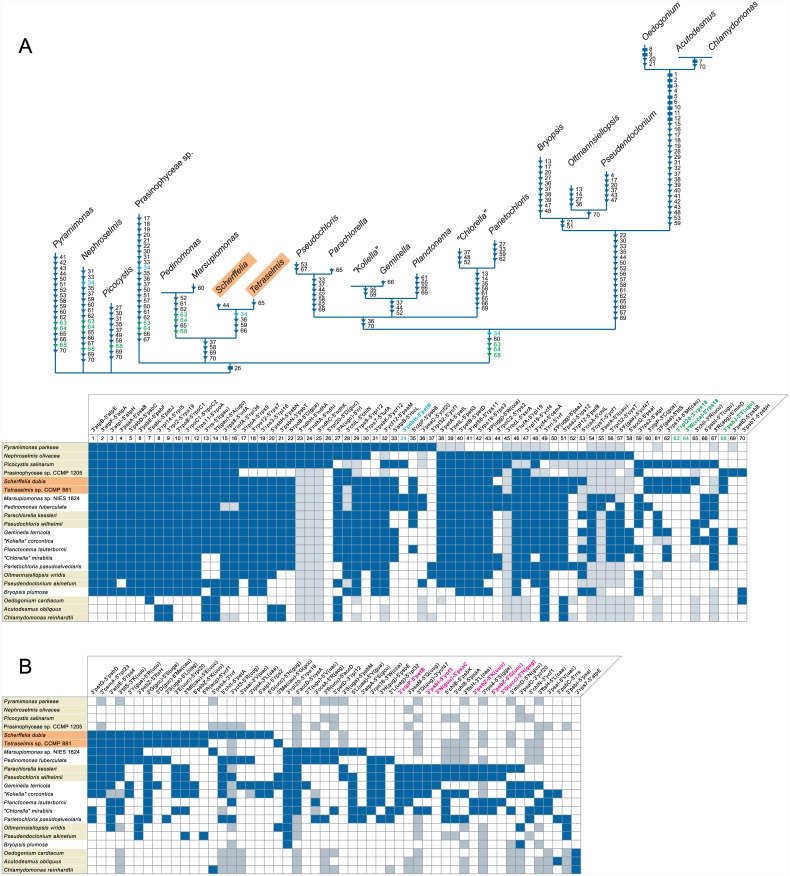
Shared gene pairs in chlorophyte chloroplast genomes. The gene pairs that are shared by at least three taxa were identified among all possible signed gene pairs in the compared genomes. The presence of a gene pair is denoted by a blue box; a gray box refers to a gene pair in which at least one gene is missing due to gene loss. (A) Retention of prasinophyte gene pairs among core chlorophytes. The tree topology shown in Fig 7 was used to map losses of prasinophyte gene pairs. The characters indicated on the branches are restricted to those involving no gene losses; the characters denoted by triangles and rectangles represent homoplasic and synapomorphic losses, respectively. The full names of the gene pairs corresponding to the character numbers are given above the distribution matrix. The three chlorodendrophycean gene pairs highlighted in green and the pedinophycean gene pair highlighted in cyan are shared exclusively with prasinophyte genomes. (B) Gain of derived gene pairs among core chlorophytes. The six gene pairs highlighted in magenta denote synapomorphic characters uniting the Chlorellales and core trebouxiophyceans. Note that seven gene pairs (3'*psaM*-5'*trnQ*(uug), 3'*trnQ*(uug)-3'*ycf47*, 5'*chlB*-5’*psbK*, 3'*chlB*-5'*psaA*, 3'*ftsH*-3'*trnL*(caa), 3’*rps4*-5’*trnS*(gga) and 3'*minD*-5'*trnN*(guu)) could not be unambiguously included in this list of synapomorphies because at least one gene in each pair is missing in some taxa. Also note that the synapomorphic signatures of all highlighted gene pairs were confirmed using a larger data set including the gene pairs of all currently available chlorophyte chloroplast genomes.

To compare the *Scherffelia* and *Tetraselmis* chloroplast gene organizations with those of other core chlorophytes, we analyzed all possible gene pairs found in the core chlorophyte genomes listed in Table 1 as well as in the cpDNAs of four prasinophytes representing distinct lineages (Fig 8). The genomes of the Chlorodendrophyceae have retained the most gene pairs from their prasinophyte ancestors, as indicated by their short branches in the cladogram of Fig 8A; they exhibit three gene pairs of prasinophyte origin that are not found in any of the other core chlorophyte lineages examined, whereas the Pedinophyceae exhibit only a single pair (Fig 8A). This observation supports the deep placement of the Chlorodendrophyceae in the inferred chloroplast trees (Figs 5–7). There is no indication, however, that this lineage forms a monophyletic group with the Pedinophyceae as we observed in the 108-gene trees (Fig 7), because no gene pairs of more recent origin unite them to the exclusion of the other core chlorophytes (Fig 8B). Likewise, the clustering of the Chlorellales and Pedinophyceae in trees inferred from the 79-gene data sets (Figs 5 and 6) is not supported by the presence of synapomorphic gene pairs uniting these lineages (Fig 8B). Conversely, there are six gene pairs that unite the Chlorellales and core trebouxiophyceans (Fig 8B), thus supporting the monophyly of the Trebouxiophyceae observed in the 108-gene trees.

## Conclusion

The chloroplast phylogenomic and structural analyses reported in this study support the notion that the Chlorodendrophyceae is an early lineage of the core Chlorophyta, although its precise placement relative to other chlorophyte lineages could not be resolved. Despite these ambiguities, our results provide a better understanding of the relationships within the core Chlorophyta by shedding light on the monophyletic/paraphyletic status of the Trebouxiophyceae. Indeed, our finding of synapomorphic gene pairs uniting the Chlorellales and core trebouxiophyceans together with the recovery of the Trebouxiophyceae as a monophyletic group in the trees inferred from the 108-gene data sets offer further evidence that the previously observed affiliation between the Pedinophyceae and Chlorellales is incorrect. As pointed out by Lemieux et al. [13], the affiliation of the latter lineages in phylogenomic analyses of chloroplast genes and proteins is likely due to improper modeling of character evolution. The finding that the chloroplast proteins of Chlorellales and Pedinophyceae share similar amino acid composition prompted these authors to suggest that the two algal groups were attracted to each other because of their similar compositional bias [13]. It is well known that heterogeneity of nucleotide or amino acid composition across lineages violates the homogeneity hypothesis of evolutionary models and leads to incorrect grouping of taxa sharing the same bias [14]. In future chloroplast phylogenomic studies, broader sampling of chlorophytes, in particular of ulvophycean lineages, as well as the use of improved models of sequence evolution might allow the construction of more robust and reliable trees. The chloroplast phylogenomic approach, however, may have limitation in its resolving power and nuclear transcriptome data might be required to resolve the radiation of core chlorophytes.

Characterized by a gene-rich IR and a SSC region devoid of any gene, the quadripartite architecture of the *Scherffelia* and *Tetraselmis* chloroplast genomes is unique among the core Chlorophyta. This unusual structure appears to have evolved by remodeling, through multiple expansions of the IR, of an ancestral core chlorophyte genome that was likely partitioned in the same fashion as extant pedinophycean and trebouxiophycean cpDNAs. These gene rearrangements occurred concomitantly with the transfer of *psbM* to the nucleus and the losses of five other protein-coding genes (*accD*, *cysA*, *cyst*, *minD*, *ycf47*) from the chloroplast genome. Following the divergence of the *Scherffelia* and *Tetraselmis* lineages, the IR underwent further expansions/contractions and gene shuffling, highlighting the dynamic evolution of this cpDNA region in the Chlorodendrophyceae.

## Supporting Information

S1 Fig Secondary structure model of the RNA species encoded by the *Scherffelia* chloroplast *rnpB* gene. The model is based on the secondary structure of the *E*. *coli* RNase P RNA, and helical regions are numbered accordingly [33]. The residues participating in the long-range P4 pairing are denoted by the brackets. The bases in boldface and italics are conserved in the *Nephroselmis olivacea* RNase P RNA [34].
(PDF)

S2 Fig Compared secondary structure models of the *Scherffelia* group II introns. (A) Consensus secondary structure of the *Scherffelia atpA* and *cemA* introns. (B) Consensus secondary structure of the *Scherffelia petA* and *petB* introns. Intron modeling was according to the nomenclature proposed for group II introns [32]. Exon sequences are shown in lowercase letters. Roman numbers specify the major structural domains. Tertiary interactions are represented by dashed lines, curved arrows and/or Greek lettering. The nucleotide positions that differ in the compared models are indicated by dots, whereas conserved base pairings are denoted by dashes. The numbers inside the variable loops and in the brackets indicate the numbers of nucleotides in these regions for the compared introns (from left to right, *atpA* and *cemA* introns in panel A, *petA* and *petB* introns in panel B). Nucleotides in boldcase letters in panel A are conserved in the group II intron identified in *Euglena myxocylindracea psbA* [53].
(PDF)

S1 Table List of all oligonucleotide primers employed in this study. (PDF)

S2 Table Sources and GenBank accession numbers of the chloroplast genomes used in the phylogenomic analyses. (PDF)
