# Supplementary figures and images for: Distinctive Architecture of the Chloroplast Genome in the Chlorodendrophycean Green Algae Scherffelia dubia and Tetraselmis sp. CCMP 881
Source: PLoS One. 2016 Feb 5;11(2):e0148934. doi: 10.1371/journal.pone.0148934 (PMC4743939; doi:10.1371/journal.pone.0148934)

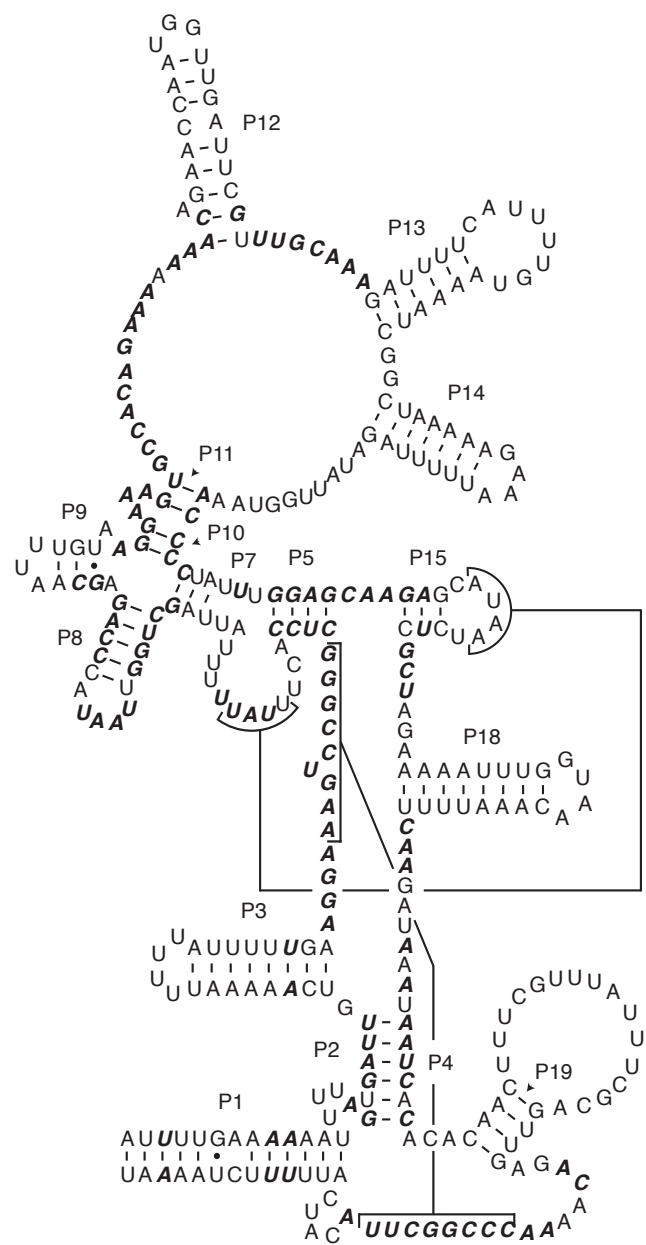

Supplement: S1 Fig — The model is based on the secondary structure of the E. coli RNase P RNA, and helical regions are numbered accordingly [33]. The residues participating in the long-range P4 pairing are denoted by the brackets. The bases in boldface and italics are conserved in the Nephroselmis olivacea RNase P RNA [34]. (PDF) [file pone.0148934.s001.pdf]

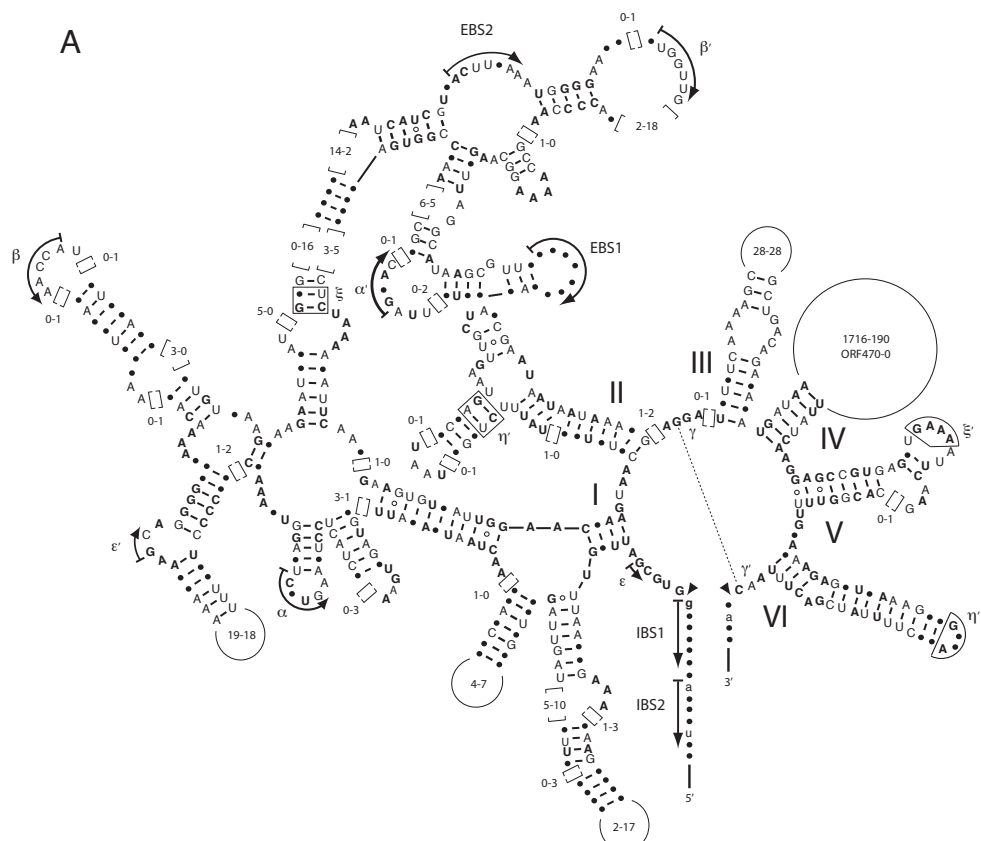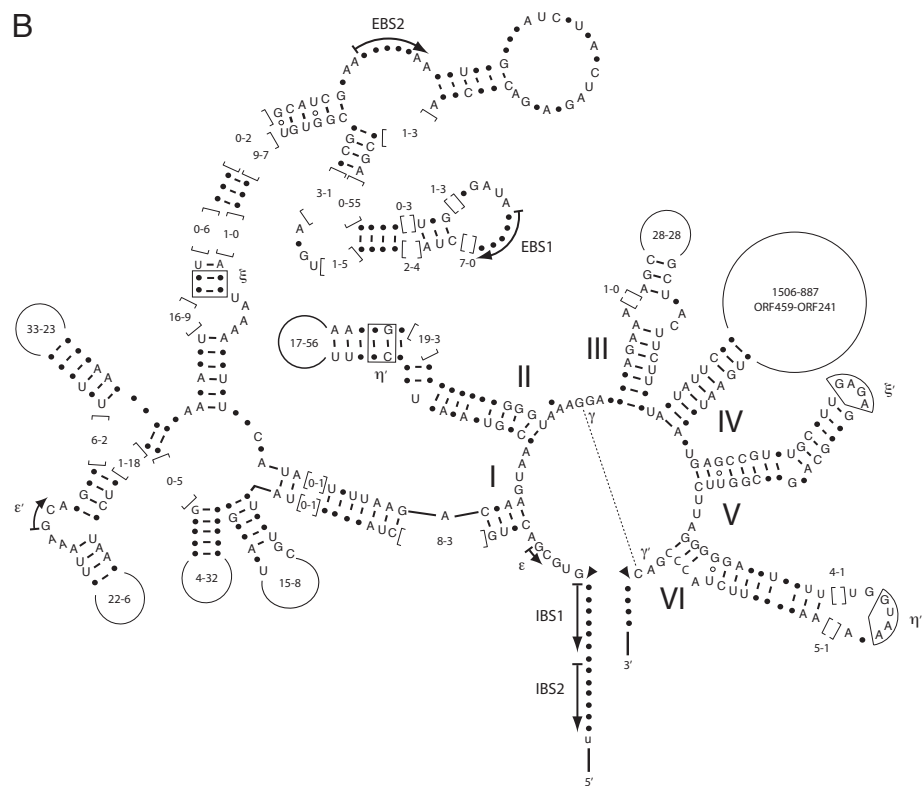

Supplement: S2 Fig — (A) Consensus secondary structure of the Scherffelia atpA and cemA introns. (B) Consensus secondary structure of the Scherffelia petA and petB introns. Intron modeling was according to the nomenclature proposed for group II introns [32]. Exon sequences are shown in lowercase letters. Roman numbers specify the major structural domains. Tertiary interactions are represented by dashed lines, curved arrows and/or Greek lettering. The nucleotide positions that differ in the compared models are indicated by dots, whereas conserved base pairings are denoted by dashes. The numbers inside the variable loops and in the brackets indicate the numbers of nucleotides in these regions for the compared introns (from left to right, atpA and cemA introns in panel A, petA and petB introns in panel B). Nucleotides in boldcase letters in panel A are conserved in the group II intron identified in Euglena myxocylindracea psbA [53]. (PDF) [file pone.0148934.s002.pdf]
