# Supplementary material for: Distinctive Architecture of the Chloroplast Genome in the Chlorodendrophycean Green Algae Scherffelia dubia and Tetraselmis sp. CCMP 881
Source: PLoS One. 2016 Feb 5;11(2):e0148934. doi: 10.1371/journal.pone.0148934 (PMC4743939; doi:10.1371/journal.pone.0148934)
Supplement: S1 Table — (PDF) [file pone.0148934.s003.pdf]

S1 Table. Oligonucleotide primers employed in this study.

Name	Sequence	Strand	Genomic position (3’ end)
***Tetraselmis* sp. CCMP 881 chloroplast genome**			
T0130Ra	CAAGCGATAAAGATTCATGAGC	-	659
T0413La	GATGGAACACCTATTGATATGG	+	3805
T0413Ra	AACATAGTGCTTGTAATTCACG	-	4376
T0356La	CCAAAAACGTTACCTACAATAG	+	14897
T0356Ra	AATGCAGTTGGACCTGTTTAAC	-	15407
T0280La	CATTTTTAACCAATTCTCTGCAGC	+	19041
T0280Ra	TTGGAGCTTATTCTGGTTTAGG	-	19777
T0873Ria	TTGTCTGATCTACCCAAATAGG	+	26148
T1492Ra	TTAATGTTGCTCGTCTTTCTGC	-	27784
T0226Ra	TGGGCATAGTATGAAAGAGATC	+	51618
T0226La	TCCATTGAGCAAAGACAGGTTC	-	52070
T0325La	ACTGGTTTGGAAAGCAATGTAC	+	56254
T0325Lb	CATATTCATTAGCCGCTATCTC	+	56551
T0326Rb	ATGGACGCATTGATAATACAGG	-	57104
T0288Ra	CTGGTACTAATGGTGTTGCTGC	+	76420
T0955Ra	TACGAATTCGACATCAGGTAGG	+	98687
T0484Ra	GAAAGTCTACCTATACAGTATAC	+	99840
T0326Ra	TATCAGGCTAGCGGAAGACTTG	+	100160
***Scherffelia dubia* chloroplast genome**			
H0647t7inva	TAACACCATCTAATTCTCCACC	+	431
H0380t3a	GCTGTTTTTGATCCTTCTGACC	-	1491
H2901t3a	CGGTGATTCAACAGCGCAATGG	+	1871
H1382t3a	TCTGCCTTACCACTTGGCGATG	+	2720
H0843t3a	CCGACCTTCCTGAATAATCCAC	-	8550
H0843t3inva	CAAAGTTAGTCCGCGGCAAAGC	+	8850
H0783t3a	TAACTCTTCCTTCTGGATCAGC	-	9880
H0227t7a	AGCAGTTGGTATTTTAGCGGCC	+	10433
H0227t3inva	TTTGAGTCTTCAGGACAAATTG	+	11091
H0227t3invb	AACTTTTAAATCTGGTTGGCCG	+	11648
H0227t3invc	TTCTGAGATTCATCGAGAATCG	+	12047
H0227t3a	ACAAAAGAATACGAAAGCCACG	-	12144
H0227t3b	TTTGACCGGAATGAGGATTTGC	-	12508
H0227t3b	TTCTACAAGCCGAAGCCATACC	-	12808
H0263t3invd	CCTAAAACGATTGGGTGATAAG	-	13124
H0263t3a	ATTGCGAATCCAAATTCGCGAG	+	13388
H0263t3a	CCAAATTCGCGAGATTTTACTC	+	13397
H0263t3invc	AACGTTCAAAAGTCCATGACTC	-	13539
H0263t3b	AGGCGTTACAACGATTTGCTGC	+	14050
H0263t3invb	TACAGAACGTAATGGGATTCAC	-	14178

H0263t3inva	CACCTGCTGGGATAGATTGAAG	-	14754
H0458t7a	CCATCACCAACTTGAAAAACAG	-	16716
H0385t7inva	TTATTTCCATACCAGTCAAGCC	+	18029
H1408t7inva	GCCCAAGATCTTAATTTTTGCC	-	18918
H1408t3a	TCTCGTTGTCCACGTCCGATTG	-	19451
H0503t7a	GTGACGTTTCAGCTTATATTCC	+	19972
H0503t7a	TTTCTGTTTCGCGTGTAGGTTC	+	20083
H0322t3inva	TTGGTGTTGATACATCTGCTGC	-	23773
H0421t7a	CTAATCGAATAGCTCGAAACGG	+	24768
H0421t3inva	GGAGCGCGACAACCATTATGAG	+	25606
H0421t3invb	CCTCGATTTAAGTCGTAAGGAC	+	26155
H0585t3invb	ATCTAAGTGATATGCTCACACG	-	26714
H0585t3inva	AAACACGAAGTTCCACGTCTTG	-	27233
H1093t7a	CGATCATTGTATGATAGGTTGC	+	30801
H1093t3inva	GCTACCAAAGATTGATAACGTG	+	31490
H0835t3inva	TATTGGCGCTTTAGACATATCC	-	31727
H0835t3a	AGCATTTTCGAAAGTAAAGCGG	-	32138
H0835t3inva	AAGTAAGAGGTTGGCTAATTCC	+	32547
H0342t7inva	AAGAGTCTCAATTGATTCGTCG	-	32973
H2165t3inva	TTAAGCGTCCAATCATTCAACC	+	36423
H2165t3invb	GCACTCCATTAGTAAAACAAGG	+	36934
H2165t3invc	TTAAAATTACTAGGACTCATTCCG	+	37591
H2165t3c	TCGTCTAGAGGCCTAGGACATC	-	37646
H2165t3invd	ACGCTTTTTTTCGCGTTCAATC	+	38144
H2165t3b	TACGTCTTACTAAATGGATAACG	-	38240
H2165t3inve	TTTACGCGAATAATAATCTGACTC	+	38655
H2165t3a	ATCCACTGCTCTACCAACTGAG	-	38791
H2165t3invf	ATACTATCATTACTGGGCATAG	+	39247
H2165t3d	TAAATTTGAAACTCCAGAGCCC	-	39288
H2165t3invg	CTCGTTTTCTTCTAATTCGTCC	+	39436
H0133t7invg	ATGAGTGTTTGTAAAAGCAGAC	-	40547
H0133t7invf	CGGATTTCTTTCAACTTAGTTCC	-	40800
H0133t3a	AAGATAAAGCTCTAGTAAATGATC	+	41117
H0133t7inve	AGGAAATTCATCCAATTGATAATC	-	41205
H0133t3b	GCTCAAAACTGCGAGAATGATC	+	41537
H0133t7invd	AGGGTAATAGGTCCACAATCTC	-	41727
H0133t3c	GTGAAGGGATTCTTTATAGTGG	+	42102
H0133t7invc	TTTCGACTAAATTTCTCTTTAGAG	-	42325
H0133t7invb	TAGGTTCCCAAGTATCGGTTTG	-	42735
H0133t7inva	TGTAAACGTTGAGCGCCTAACC	-	43204
H0765t3a	GGCACTATGAGTGTTTCGACTG	-	47559
H0765t3inva	CTGATTACCAAGTACTAGACAC	+	48039
H0765t3invb	GATCGTTTCTGTTTCGTTGCAG	+	48605
H0308t7invb	TCTTTCCAAACTTCACAGGCAG	-	49306
H0308t7inva	ACTGAAGGTGAGGGTTCGATTC	-	49908
H0308t3a	AAGCCGGGTTTTGTCAACTGCG	-	50825

H1019t7inva	AATGGGCTAGAAAACGCCATGG	+	51155
H2613t3a	GTATAATAGAGATTGTGAGCCTC	+	52026
H0101t3inva	TCGGATTTGAACCAACGTCGTC	-	52768
H1113t3a	TAGGCGTCGCCAGCGGATTTAC	-	53753
H1113t3inva	TGGTTCGAATCCAGGCAGGAGC	+	53900
H1294t3inva	TACCAGAAATTCCAGTTGTCGC	-	54810
H0168t3a	AGGTTATGCTGTACGCATGAAC	-	55454
H1019t7a	ATGGCGTTTTCTAGCCCATTTC	-	57130
H1019t7inva	CCTCTTATTCAAATTCCTCAGC	+	57281
H1292t7inva	TAACAATAACTTCTGGGCCAGC	-	58047
H0538t7a	ACCAGTTATATTGGATTACTTGC	+	58338
H1292t3a	TTGTATTTTCAGACCGTGCAAC	-	58910
H2437t7a	AACCTGTTTTATCAATGCGTCC	+	61904
H0285t3inva	GTAGCAAAACGAACGGCTCGAC	+	65735
H0285t3inva	GCCAATGGCTTATAGAAGAATAG	+	65806
H0113t7invb	TCCTTATTGCTTCAACATCTGC	+	68877
H0113t7inva	TTAGAGAACCACCACGTATTGC	+	69206
H0647t3inva	TCTCGTAGTTCTTTTGAGGGAG	+	69802
H0647t3inva	AATATGGTAAAGTTGCTCCAGG	+	70265
H0452t3a	AATGCTTCAAATGTGCCTCCGG	+	75459
H1211t3a	CCCATGCAAACCCATTAGAAGC	+	88988
H0360t3invb	GACAGACGGCGAAACGAACCAG	+	100228
H0360t3inva	GCGATAAATGGCTAGAAAGACG	+	100520
H0360t3invc	GATGTTGGAATTACAGCACCAG	+	101048
H0360t3invd	CCAGCTCTTTGAATTTTCAAAGCC	+	101341
H0360t3invea	ATTTCGCTTTTGTTAAGGGAGG	+	101912
H0360t3b	CATCGCAGACGTCCTTAGGTTC	-	102130
H0360t3inveb	TGGTGCTGTAGAGGTAGAACCG	-	104524
H0360t3a	GCTTTGAAAATTCAAAGAGCTGGG	+	105003
H2013t3inva	AATCGTCATTAACTCCTCTCGG	+	135452
