# Supplementary material for: Distinctive Architecture of the Chloroplast Genome in the Chlorodendrophycean Green Algae Scherffelia dubia and Tetraselmis sp. CCMP 881
Source: PLoS One. 2016 Feb 5;11(2):e0148934. doi: 10.1371/journal.pone.0148934 (PMC4743939; doi:10.1371/journal.pone.0148934)
Supplement: S2 Table — (PDF) [file pone.0148934.s004.pdf]

S2 Table. Sources and GenBank accession numbers of the chloroplast genomes used in the phylogenomic analyses.

Taxon	Source [a]	GenBank accession
**Prasinophyceae**		
Mesostigma viride	NIES 296	NC_002186
Chlorokybus atmophyticus	SAG 48.80	NC_008822
Prasinococcus sp.	CCMP 1194	KJ746597
Prasinophyceae sp.	MBIC 106222	KJ746602
Prasinoderma coloniale	CCMP 1220	NC_024817
Pyramimonas parkeae	CCMP 726	NC_012099
Monomastix sp.	OKE-1	NC_012101
Ostreococcus tauri	OTTH0595	NC_008289
Micromonas sp.	RCC299	NC_012575
Nephroselmis olivacea	NIES 484	NC_000927
Nephroselmis astigmatica	NIES 252	NC_024829
Pycnococcus provasolii	CCMP 1203	NC_012097
Picocystis salinarum	CCMP 1897	NC_024828
Prasinophyceae sp.	CCMP1205	KJ746601
**Pedinophyceae**		
Marsupiomonas sp.	NIES 1824	KM462870
Pedinomonas tuberculata	SAG 42.84	NC_025530
Pedinomonas minor	UTEX LB 1350	NC_016733
**Chlorodendrophyceae**		
Tetraselmis sp.	CCMP 881	KU167097
Scherffelia dubia	SAG 17.86	KU167098
**Chlorellales**		
Dicloster acuatus	SAG 41.98	NC_025546
Parachlorella kessleri	SAG 211-11g	NC_012978
Pseudochloris wilhelmii	SAG 1.80	NC_025547
Marvania geminata	SAG 12.88	NC_025549
Chlorella vulgaris		NC_001865
Chlorella variabilis	NC64A	NC_015359
**Core Trebouxiophyceae**		
Koliella corcontica	SAG 24.84	NC_025536
Geminella terricola	SAG 20.91	NC_025542
Geminella minor	SAG 22.88	NC_025544
Gloeotilopsis sterilis	UTEX 1704	NC_025538
Oocystis solitaria	SAG 83.80	FJ968739 [b]
Planctonema lauterbornii	SAG 68.94	NC_025541
Pleurastrosarcina brevispinosa	UTEX 1176	KM462875 [b]
Neocystis brevis	CAUP D802	NC_025535
Stichococcus bacillaris	UTEX 176	NC_025527
Prasiolopsis sp.	SAG 84.81	KM462862
“Chlorella” mirabilis	SAG 38.88	NC_025528
Koliella longiseta	UTEX 339	NC_025531
Pabia signiensis	SAG 7.90	NC_025529
Parietochloris pseudoalveolaris	UTEX 975	NC_025532
Leptosira terrestris	UTEX 333	NC_009681
Xylochloris irregularis	CAUP H7801	NC_025534

Microthamnion kuetzingianum	UTEX 318	NC_025537
Fusochloris perforata	SAG 28.85	NC_025543
Trebouxia aggregata	SAG 219-1d	EU123962-EU124002 [b]
Myrmecia israelensis	UTEX 1181	NC_025525
Lobosphaera incisa	SAG 2007	NC_025533
Dictyochloropsis reticulata	SAG 2150	NC_025524
Watanabea reniformis	SAG 211-9b	NC_025526
Choricystis minor	SAG 17.98	NC_025539
Botryococcus braunii	SAG 807-1	NC_025545
Elliptochloris bilobata	CAUP H7103	NC_025548
Trebouxiophyceae sp.	MX-AZ01	NC_018569
Coccomyxa subellipsoidea	C-169	NC_015084
Paradoxia multiseta	SAG 18.84	NC_025540
**Ulvophyceae, Ulvales**		
Oltmannsiellopsis viridis	NIES 360	NC_008099
Ulva sp.	UNA00071828	KP720616
Pseudendoclonium akinetum	UTEX 1912	NC_008114
**Ulvophyceae, Bryopsidales**		
Tydemania expeditionis		NC_026796
Bryopsis hypnoides		NC_013359
Bryopsis plumosa	West4718	NC_026795
**Chlorophyceae**		
Oedogonium cardiacum	SAG 575-1b	NC_011031
Floydiella terrestris	UTEX 1709	NC_014346
Stigeoclonium helveticum	UTEX 441	NC_008372
Schizomeris leibleinii	UTEX LB 1228	NC_015645
Mychonastes jurisii	SAG 37.98	KT625411
Acutodesmus obliquus	UTEX 393	NC_008101
Carteria sp.	SAG 8-5	KT625419
Dunaliella salina	CCAP 19/18	NC_016732
Chlamydomonas moewusii	UTEX 97	EF587443-EF587503 [b]
Chlamydomonas reinhardtii		NC_005353
Volvox carteri f. nagariensis	UTEX 2908	GU084820

[a] Most taxa originate from the culture collections of algae at the University of Goettingen (SAG), the University of Texas at Austin (UTEX), the Provasoli-Guillard National Center for Marine Algae and Microbiota (CCMP), the National Institute of Environmental Studies in Tsukuba (NIES), the Scottish Association for Marine Science in Oban (CCAP), the National Institute of Technology and Evaluation Biological Resource Center in Tokyo (MBIC), the Biological Station of Roscoff (RCC), and Charles University in Prague (CAUP).

[b] The chloroplast genome sequences of *Oocystis solitaria, Pleurastrosarcina brevispinosa, Trebouxia aggregata* and *Chlamydomonas moewusii* are not complete.
